# Impact of patron saint festivities on cholera in three communes in Haiti

**DOI:** 10.1186/s12889-020-09601-9

**Published:** 2020-10-01

**Authors:** Kenny Moise, Aude Mélody Achille, Doudou Batumbo, Bertiny Bourdeau, Stanislas Rebaudet, Gérald Lerebours, Jean Hugues Henrys, Christian Raccurt

**Affiliations:** 1 Equipe de Recherche sur les Maladies Infectieuses, Parasitaires et Tropicales, Université Quisqueya, 218 Avenue Jean-Paul II, Port-au-Prince, 6110 Haiti; 2 Unité de Formation et de Recherche sur l’Eco-épidémiologie des Maladies Infectieuses, Université de Kinshasa, Kinshasa, République Démocratique du Congo; 3 APHM, Aix Marseille Univ, INSERM, IRD, Hôpital Européen, SESSTIM, Marseille, France

**Keywords:** Cholera, Patronal festivities, Mass gatherings, Haiti

## Abstract

**Background:**

Religious pilgrimages are among the anthropogenic factors known to be associated with the transmission of diarrheal diseases, such as cholera. This ecological study aimed to describe the evolution of cholera and assess the relationship between the implementation of the ‘coup de poing’ strategy during the patron saint festivities and the incidence of cholera in the three communes of Cabaret, Carrefour, and Croix-des-Bouquets in Haiti in 2017.

**Methods:**

An epidemiological curve was produced to illustrate the evolution of cholera at the communal level. Generalized linear models assuming a Poisson distribution were used to weight the annual cholera incidence of communal sections against variables such as the number of patronal festivities, population density and annual precipitation rates. The number of cases in the week of the festivity as well as one and 2 weeks later was weighted against patronal festivities and weekly precipitation rates.

**Results:**

In total, 3633 suspected cholera cases were continuously reported in three communes in Haiti (Cabaret, Carrefour, Croix-des-bouquets) during the 52-epidemiological week period in 2017. After controlling for rainfall and population density, the implementation of the ‘coup de poing’ strategy during the patron saint festivities was associated with a significant reduction in cholera incidence of 57.23% [PR = 0.4277 (97.5% CI: 0.2798–0.6193), *p* = 0.0000244]. The implementation of the strategy was associated with a reduction in cholera incidence of 25.41% 1 week following patronal festivities.

**Conclusion:**

This study showed a continuous presence of cholera in three communes in Haiti in 2017 and an association between the implementation of the ‘coup de poing’ strategy during patronal festivities and a reduction in cholera incidence. The findings imply that the multi-partner ‘coup de poing’ strategy may have contributed to the reduced cholera incidence following patron saint festivities and in Ouest department in Haiti in 2017.

## Background

During a cholera epidemic, the human-to-human transmission such as that noted in large gatherings, without involving the aquatic environment becomes the most important route of transmission [1, 2]. In Haiti, the celebration of local patron saints represents a highlight of cultural and religious life and involves large movements of thousands of pilgrims at fixed periods over the year. In 2014, the mishandling of a patron saint festivity in the Artibonite department was cited as the potential cause of an outbreak that spread to the rest of the country [3]. Since the incidence of cholera in 2016 was increased at the national level compared with incidence rates in 2014, six communes, including Cabaret, Carrefour and Croix-des-Bouquets, have been identified by the Department of Epidemiology, Laboratories and Research (DELR) as contributors to recurrent outbreaks in the remainder of the country at key time periods, such as periods of time following patron saint festivities [4].

A multi-partner “coup de poing” strategy has been launched in July 2017 to respond to the cholera epidemic in the Ouest department, mainly in these communes in order to reinforce the alert-response strategy upstream of mass gathering events such as patronal festivities [5]. Under the leadership of the Ministry of Health and the National Directorate of Drinking Water and Sanitation (French acronym DINEPA), it entailed reinforced epidemiologic surveillance, investigations, institutional management, community response and communication.

During the operation, 26 mobile teams have been deployed among which 10 response teams and 16 community outreach teams in charge of door-by-door awareness and follow-up on interventions. As of November 2017, the teams responded to 1102 alerts. In total, 8929 sanitary kits and 644,123 oral rehydration solutions were distributed, 14,058 water point decontaminations conducted, 69 chlorination points and 17 hand washing points installed, 78 mass awareness sessions organized in marketplaces, churches and schools, and 10,744 flyers containing awareness messages distributed reaching 1110 individuals. However, vaccination was not involved in the intervention package. Although an oral cholera vaccine (OCV) coverage was not determined for each communal section prior to the intervention, the Ouest department showed a 44% of two-dose coverage in 2014 [6]. Previous OVC campaigns in Haiti were limited. A pilot project was conducted in a rural area in the Artibonite department in 2012, followed by targeted campaigns in specific communes in the North in 2013, South in 2016 and Centre department in 2013 and 2017 [7–9].

As of the 32nd epidemiological week of 2020, no reported cases have been biologically confirmed since the 4th week of 2019 [10]. However, the long-term phase of the 10-year plan to eliminate cholera has been launched in 2019 with an emphasis on access to clean water and sanitation. This study aimed to describe the evolution of cholera and assess the relationship between the implementation of the ‘coup de poing’ strategy during the patron saint festivities and the incidence of cholera in the three communes of Cabaret, Carrefour, and Croix-des-Bouquets in 2017.

## Methods

### Data source

A cholera case was defined as any patient presenting to a health facility after developing acute diarrhoeal disease with or without vomiting [4]. Cases of cholera during the period from January 1st, 2017, to December 31st, 2017, were obtained from the electronic databases maintained by the Ouest departmental health direction, which is updated weekly from the paper registry used by the clinical staff of the Cholera Treatment Centers. The number and date of occurrence of patron saint holidays per communal section over the period were collected from an online registry of the Ministry of Agriculture, Natural Resources and Rural Development (MANRRD) (Table 1), and the Catholic Church calendar [11]. We collected population counts and density data from the Haitian Informatics and Statistics Institute (HISI) and daily precipitation data from the National Aeronautics and Space Administration (NASA) Giovanni data analysis system [12, 13]. The components and activities of the “coup de poing” strategy were retrieved from an unpublished report of DINEPA of the ‘coup de poing’ operation (Table 2).

**Table 1:** Patron Saint Festivities at communal sections level in 3 communes in Haiti

Communes	Communal sections	Patron saints	Date of occurrence
Cabaret	1^ère^ section Boucassin	Saint Agnès	July 16
Cabaret	2^ème^ section Boucassin	Mont Carmel	July 16
Cabaret	2^ème^ section Boucassin	Saint Joseph	March 19
Cabaret	3^ème^ section Source Matelas	Saint Gérard	October 6
Cabaret	4^ème^ section Fonds des Blancs	Saint Michel	September 29
Croix-des-Bouquets	1^ère^ section Varreux	Sainte Thérèse	October 1
Croix-des-Bouquets	2^ème^ section Varreux	Marie Madeleine	July 22
Croix-des-Bouquets	2^ème^ section Varreux	Michel-Ange	September 30
Croix-des-Bouquets	3^ème^ section Petit Bois	Saint Antoine	June 3
Croix-des-Bouquets	4^ème^ section Petit Bois	Notre Dame du Rosaire	June 24
Croix-des-Bouquets	4^ème^ section Petit Bois	Notre-Dame du Perpétuel Secours	October 7
Croix-des-Bouquets	5^ème^ section Petit Bois	Notre Dame de Lamercie	September 24
Croix-des-Bouquets	6^ème^ section Belle Fontaine	Notre Dame Deslourdes	February 11
Croix-des-Bouquets	9^ème^ section Crochus	Sainte Marie joseph	February 4
Croix-des-Bouquets	10^ème^ section des Orangers	Sainte Geneviève	January 3
Croix-des-Bouquets	10^ème^ section des Orangers	Sainte Thérèse de l’Enfant Jésus	October 3
Carrefour	3^ème^ section Taïfer	Sainte Croix	September 14
Carrefour	7^ème^ section Lavalle	Saint Joseph	March 19
Carrefour	7^ème^ section Lavalle	Sainte Thérèse de l’Enfant Jésus	October 1
Carrefour	9^ème^ section Bizoton	Mont Carmel	July 16
Carrefour	10^ème^ section Thor	Saint Charles	November 4
Carrefour	10^ème^ section Thor	Notre-Dame du Perpétuel Secours	June 27
Carrefour	11^ème^ section Rivière Froide	Sainte-Thérèse de l’Enfant Jésus	October 1

Source: Ministry of Agriculture, Natural Resources and Rural Development, 2008. Community survey, Definitive results. http://agriculture.gouv.ht/statistiques_agricoles/EnqueteCommunautaire/documents/DEP05.html. Published online in 2008. Accessed on January 24, 2019

**Table 2:** Components and activities of the ‘coup de poing’ strategy deployed in the Ouest department in Haiti, 2017

Components	Activities
Coordination	- 55 situation room meetings
Epidemiologic surveillance	- Daily action-oriented epidemiologic analysis
Investigation	- More than 7 multisectoral and multi-partnered investigations
Care delivery	- 30 supervision visits of Diarrheal Treatment Centers - 10 follow-up visits - Training of 20 nurses, 7 nursing assistants and 10 hygienists - Installation and rehabilitation of equipment on 3 outbreak sites by partner NGOs - Supply of treatment centers with materials and establishment of a contingency stock - Transport of specimen to the National public health laboratory by partner NGOs
Community response and water, sanitation and hygiene	- Deployment of 26 mobile teams - Distribution of 8929 sanitary kits - Distribution of 644,123 oral rehydration solutions - Operation of 14,058 water point decontaminations - Installation of 69 chlorination points - Installation of 17 hand washing points - Organization of 78 mass awareness sessions in marketplaces, churches and schools - Distribution of 10,744 flyers containing awareness messages
Communication	- Training of 401 focal points in local town halls - Training of 108 marketplace directors, 155 grassroot organizations directors and 136 funeral directors - 43 street theater performances reaching 4200 individuals - 30 public transportation debates reaching 7350 individuals - Distribution of T-shirts and USB with awareness messages reaching 9600 individuals - 6 workshops targeting 210 religious leaders - Involvement of 85 churches reaching 17,000 individuals

Source: DINEPA, 2017. [Report] Results of the ‘coup de poing’ strategy against cholera, Ouest department, Haiti

### Data analysis

For spatial analysis, we examined the number of patronal festivities, annual case count, population count, population density and annual precipitation rates per communal section. Incidences per 100 inhabitants were calculated by dividing the case counts by population size. The data were imported in R® software (version 3.6.0. The R Foundation for Statistical Computing. April 26, 2019). Using a generalized linear model (glm) and assuming a Poisson distribution, we weighted the annual cholera incidence against the number of festivities per communal section, annual precipitation rate and population density. Since the ‘coup de poing’ data were collected at the communal level, while our analysis was conducted at a finer scale, we used the patronal festivities as a proxy to assess the relationship between the implementation of the strategy and cholera incidence.

The following formula was used:

- glm (formula = Incidence ~ Festivity + Density + Rain + (1|section) + (1|commune),
  family = Poisson, data = data)

For temporal analysis, we distributed the number of patronal festivities, weekly precipitation rate and case count per epidemiological week (W0). Case counts in communal sections were also distributed for the 2 subsequent weeks (W1 and W2, respectively). A glm was used to assess the weight of case count at W0, W1 and W2 against number of patronal festivities and weekly precipitation rates. The following formulas were used:

- glm (formula = Case.W0 ~ Festivity + Rain + (1|section) + (1|commune), family =
  Poisson, data = data 1)
- glm (formula = Case.W1 ~ Festivity + Rain + (1|section) + (1|commune), family =
  Poisson, data = data 1)
- glm (formula = Case.W2 ~ Festivity + Rain + (1|section) + (1|commune), family =
  Poisson, data = data 1)

**Fig. 1:**
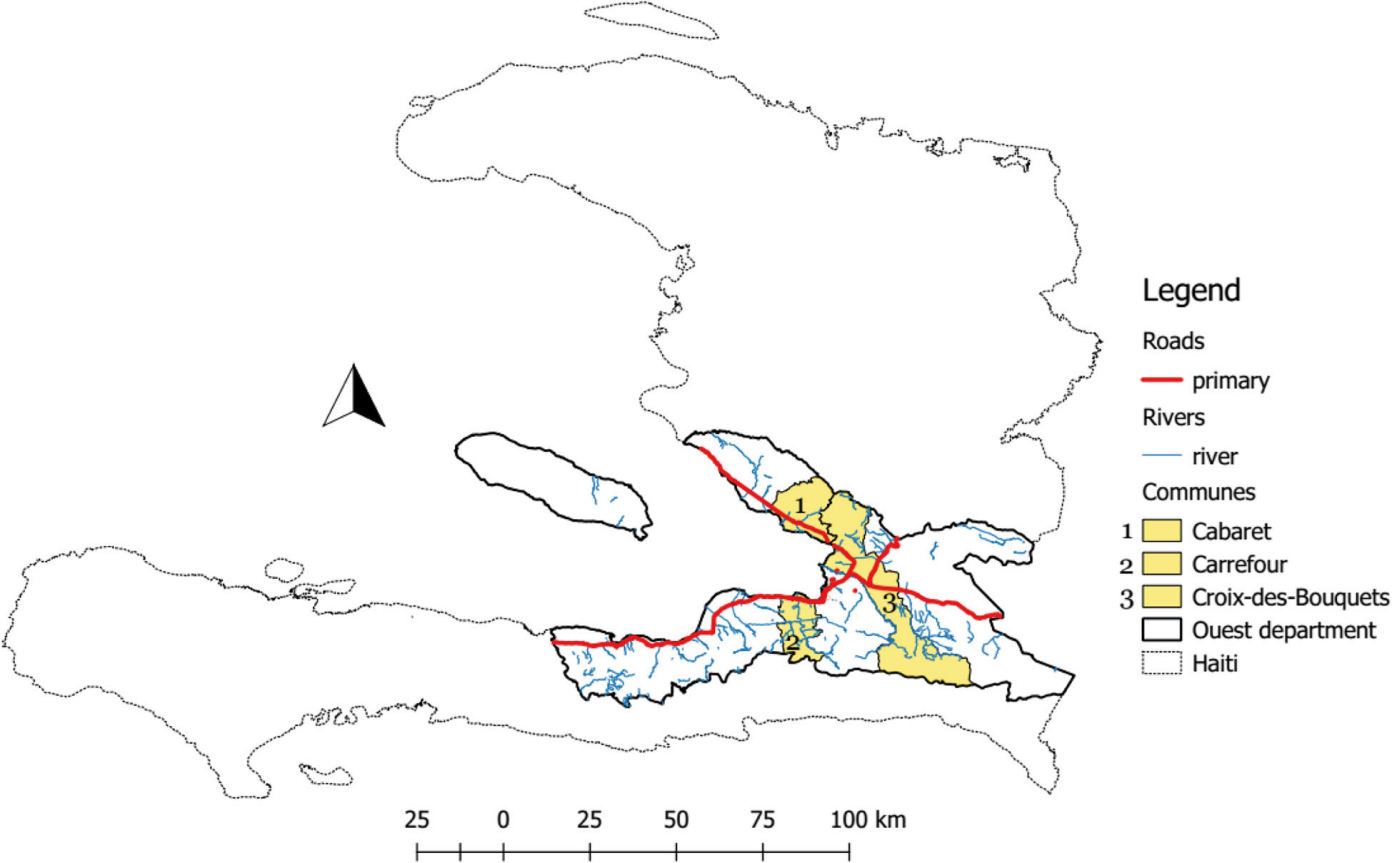
Geographic location of Cabaret, Carrefour and Croix-des-bouquets in regards to main roads and presence of rivers. National Center for Geospatial Information, 2017. Available online www.haitidata.org

For the final analysis, we combined the data of the three communes on the basis that they have similar characteristics in terms of type of land, presence of rivers and main roads and having been identified by the DELR as cholera-persistent communes (Fig. 1).

## Results

**Fig. 2:**
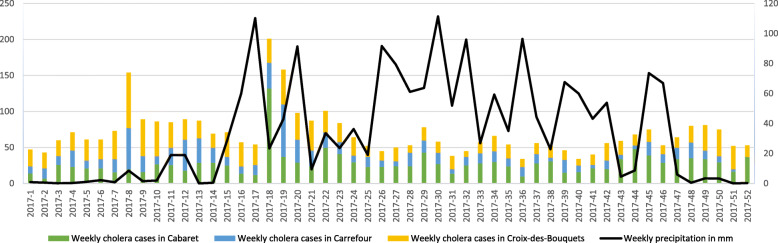
Evolution of cholera in Cabaret, Carrefour and Croix-des-Bouquets in Haiti per epidemiological week in 2017. Number of cholera cases in Cabaret, Carrefour and Croix-des-bouquets and precipitation rates per epidemiological week in mm in 2017

In 2017, Haiti reported a total of 13,681 cases of cholera to the World Health Organization among which 3 633 suspected cholera cases were recorded in Cabaret, Carrefour and Croix-des- Bouquets [14]. The attack rates were 211.44, 17.31 and 52.28 per 10,000 inhabitants respectively. The epidemiological curve shows a persistence of cholera throughout the year, with all three communes having reported cases during all 52 epidemiological weeks of 2017, and noteworthy peaks at the 8th, 18th and 19th epidemiological weeks (Fig. 2). After controlling for rainfall and population density, the implementation of the ‘coup de poing’ strategy during the patron saint festivities was associated with a significant reduction in annual cholera incidence of 57.23% [PR = 0.4277 (97.5% CI: 0.2798–0.6193), *p* = 0.0000244] in communal sections of the three communes combined.

At W0, precipitation rate [PR = 1.0000 (97.5% CI: 0.9994–1.0007), *p* = 0.828] and the implementation of the ‘coup de poing strategy’ during patronal festivities [PR = 0.9638 (97.5% CI: 0.7903–1.1617), *p* = 0.707] were not associated with cholera incidence.

At W1, the implementation of the ‘coup de poing’ strategy during patronal festivities [PR = 0.7458 (97.5% CI: 0.5943–0.9217), *p* = 0.0087] was significantly associated with a reduction in cholera incidence of 25.41% in communal sections of the three communes combined, after controlling for rainfall [PR = 1.0022 (97.5% CI: 1.0016–1.0028), *p* = 0.00000000000127].

At W2, precipitation rate [PR = 1.0030 (97.5% CI: 0.8589–1.2504), *p* = 0.002* 10^-13^] and the implementation of the ‘coup de poing’ strategy during patronal festivities [PR = 1.0420 (97.5% CI: 1.0024–1.0036), *p* = 0.667] were not associated with cholera incidence (Table 3).

**Table 3:** Model parameters and Prevalence Ratios of the Generalized Linear Model selected for cholera cases following patronal festivities at W0, W1 and W2 in communal sections, Haiti, 2017*

Characteristic	Coefficient estimate	Standard error	Z value	Pr(>|t|)	Prevalence Ratio	CI (2. 5-97.5) %
Intercept 2017	−55.93	30.60	−1.83	0.07	5.11* 10^-25^	2.40* 10^-52^–3.86
Patronal festivities in 2017	−8.49* 10^-1^	2.01* 10^-1^	− 4.22	2.44* 10^-5^ ***	0.43	2.80* 10^-1^–0.62
Density in 2017	−2.09* 10^-4^	3.37* 10^-5^	−6.20	5.83* 10^-10^ ***	1.00	10.00* 10^-1^–1. 00
Precipitations in 2017	2.97* 10^-2^	1.52* 10^-2^	1.95	0.05	1.03	1.00–1.06
Intercept W0	1.11	0.02	64.27	< 2* 10^-16^ ***	4.10	3.93–4.28
Patronal festivities at W0	−0.04	0.10	−0.38	0.71	0.96	0.79–1.16
Precipitations at W0	0.78* 10^-5^	3.60* 10^-4^	0.21	0.83	1.00	1.00–1.00
Intercept W1	1.33	0.02	60.19	< 2* 10^-16^ ***	3.79	3.63–3.96
Patronal festivities at W1	−0.29	0.11	−2.62	1.871* 10^-2^ **	0.75	0.59–0.92
Precipitations at W1	0.02* 10^-1^	0.03* 10^-2^	7.10	1.27e-12 ***	1.00	1.00–1.00
Intercept W2	1.30	0.02	57.46	< 2* 10^-16^ ***	3.65	3.50–3.82
Patronal festivities at W2	0.04	0.10	0.43	0.67	1.04	0.86–1.25
Precipitationss at W2	0.03* 10^-1^	0.31* 10^-3^	9.74	< 2* 10^-16^ ***	1.00	1.00–1.00

*Coefficient estimate, regression coefficients (for discrete variables, their exponential gives the Odds ratio); z value, value of the t distribution; Pr(>|t|), probability of the null hypothesis of a coefficient estimate not statistically different from zero; CI, confidence interval; intercept, average number of cases

## Discussion

Studies and reports have pointed out the relationship between religious pilgrimages and cholera outbreaks, such as the outbreak in Senegal between 2004 and 2006 [15, 16]^.^ In a previous study in 2014, Gazin observed a sudden increase in the number of cholera cases in Saint-Michel de l’Attalaye, in Haiti’s Artibonite department, at approximately the same time as the local patron saint festival in a nearby locality, 1 year after the launch of the National Plan for the Elimination of Cholera in Haiti [3]**.** Cholera travels with humans. On the other hand, environmental factors such as high population density and rainy seasons are known risk factors for increased cholera incidence in the Democratic Republic of Congo and other African countries [2, 17]. However, our study showed that the incidence of cholera decreases significantly a week after patronal festivities in communes of Cabaret, Carrefour and Croix-des-bouquets. This finding suggests that the “coup de poing” strategy, which was deployed upstream of mass gatherings, may have contributed in mitigating the risk of cholera outbreaks in 2017.

Lorraine Williams et al. studied the role of perceived risk in reducing cholera vulnerability in Mozambique, depicting it as a motivator in risk management approaches [18]. As they emphasized, periods perceived to be of greater risk for cholera transmission were associated with increased vigilance and risk reduction measures. Based on the experience from the outbreak described by Gazin, the concept of perceived risk might play a role in our observations, amplified by the important number of awareness initiatives conducted and individuals reached during the “coup de poing” strategy; however, more research is needed to assess the perception of risk towards infectious diseases among rural populations in Haiti during periods of patronal festivities. Furthermore, we also assessed the prevention and response strategy in relation to cholera outbreaks in Haiti.

In the context of the elimination plan, a case-area targeted rapid response strategy, that was first implemented in 2013, involves the Haitian Ministry of Health, DINEPA and international partners such as Pan-American Health Organization and United Nations Children’s Fund [19]. Various preventive and alert-response measures were thoroughly deployed from the launch in 2017 to respond to cholera alerts in a timely fashion. Patronal festivities in communal sections are occasions for which the alert-response system should be intensified in order to treat cases and prevent outbreaks. Community outreach and educational activities in areas near cases were used to reinforce the management of cases. An estimation of the effectiveness of such case-area targeted interventions in the Center department between 2015 and 2017 has shown positive outcomes in order to mitigate and shorten cholera outbreaks [20].

Regarding rainfall and population density, in our study they did not influence the annual incidence of cholera or the incidence 1 week following patronal festivities. However, studies have previously shown a significant association between seasonal rainfall and cholera [21]. Hurricane Matthew, which landed in Haiti in October 2016, was followed by increased number of reported cholera cases [22]. Marisa Eisenberg et al. also showed that increased cholera incidence was associated with cumulative precipitation greater than 30 mm 4-7 days before infections were reported [23]. However, in the context of patronal festivities, not all communal sections experienced high rates of precipitation in the week preceding the gathering events, as the events were not all held in rainy seasons, the mean rate of precipitation being 38.78 mm. A study has identified a link between cholera and social factors such as population density in vulnerable areas [24]. Nonetheless, communal sections in Haiti are largely unurbanized, therefore the incidence of cholera might be associated with different factors such as the practice of latrine sharing [25].

### Limitations and perspectives

Limitations of this study are attributable to the quality of the data recorded in the source databases and then entered into our database, specifically with regard to the origin of the patients. The origin of the patients was not recorded in all cases. In addition, the population densities of the communal sections used in this analysis are dated to 2015, based on the latest HISI estimates. These data may not reflect the current situation in the communal sections. Furthermore, as there is no national registry of interventions, data regarding cholera interventions prior to the ‘coup de poing’ strategy could not be integrated in the model. Based on our findings, further investigations should emphasize the impact of interventions following patronal festivities on cholera incidence.

We attempted to control for failure of the surveillance system during the study period using the surveillance completion data. However, this data is only collected at the departmental level (first administrative level) and could not be integrated in our model. It is likely that our observations might be due to decreased cholera case reporting from the cholera treatment centers and decreased health care attendance as health care workers and patients focus on the festivities.

We also were not able to assess the relationship with particular interventions of the ‘coup de poing’ strategy since data were collected at the communal level. However, the patronal festivities are the best proxy to the extent of our knowledge. This emphasizes the need to collect intervention data systematically at all administrative levels. Our study being inaugural, it opens doors to further investigations of spatial and temporal dynamics of cholera in regards to preventive interventions.

## Conclusion

Sociocultural factors contribute to the human-to-human transmission of cholera. In 2017, the cholera epidemic showed a persistent pattern in three communes in the Ouest department in Haiti. In this study, our model revealed a statistically significant association between the implementation of the ‘coup de poing’ strategy during patronal festivities and a reduction in cholera incidence. The findings in this study suggest that that the multi-partner ‘coup de poing’ strategy may have contributed to the reduced cholera incidence during patron saint festivities and in Ouest department in Haiti 2017.

## Data Availability

The datasets used and/or analyzed during the current study are available from the corresponding author on reasonable request. The Ouest departmental health direction database is close to public access. We obtained administrative permission to access and use it for this study. The MANRRD (http://agriculture.gouv.ht/statistiques_agricoles/EnqueteCommunautaire/documents/DEP05.html.), HISI (https://web.archive.org/web/20151106110552/http://www.ihsi.ht/pdf/projection/Estimat_PopTotal_18ans_Menag2015.pdf) and NASA (https://giovanni.gsfc.nasa.gov/giovanni/) data are available online and public access is open.

## Abbreviations

DELR: Department of Epidemiology, Laboratories and Research
DINEPA: Direction National d’Eau Potable et d’Assainissement
GLM: Generalized Linear Model
HISI: Haitian Informatics and Statistics Institute
MANRRD: Ministry of Agriculture, Natural Resources and Rural Development
NASA: National Aeronautics and Space Administration
